# Naturopathic Treatment of Grade III Oligodendroglioma With Progression to Grade IV Isocitrate Dehydrogenase (IDH)-Mutant Astrocytoma and the Development of Spinal Gliomatosis

**DOI:** 10.7759/cureus.45526

**Published:** 2023-09-19

**Authors:** Kristina F Terrani, Conner D Reynolds, Samuel N Rogers

**Affiliations:** 1 Diagnostic Radiology, University of Arizona College of Medicine - Tucson, Tucson, USA

**Keywords:** mri, sequential imaging, spinal gliomatosis, glioblastoma, astrocytoma

## Abstract

Primary intracranial gliomas are a heterogeneous class of lesions that rarely metastasize. Even more infrequently, they may spread caudally into the spinal cord causing spinal gliomatosis. In this case, we discuss an 18-year-old male patient with a diagnosis of grade IV astrocytoma with spinal gliomatosis, specifically detailing the radiographic progression of the disease over 38 months. We also discuss the significance of the change in the WHO classification of central nervous system tumors, as this patient’s survival duration is inconsistent with the low survival rates expected of glioblastoma, and rather more consistent with a grade IV astrocytoma.

## Introduction

Glial cell tumors are a heterogeneous entity comprising about 60% of primary central nervous system (CNS) neoplasms, with astrocytomas representing their most common glioma subtype [1]. Their classification is closely tied to histologic features and clinical outcomes. More recently, schemes have also begun incorporating molecular characteristics to enhance prognostic accuracy and guide therapy. Previously, grade four astrocytomas were classified as glioblastoma multiforme. However, the 2021 World Health Organization (WHO) classification of central nervous system tumors now separates tumors by the presence of isocitrate dehydrogenase (IDH) mutations, distinguishing IDH-mutant astrocytomas from IDH-wildtype glioblastomas [2].

Among these high-grade gliomas, IDH-mutant astrocytomas are much rarer, making up only about 10% of grade IV gliomas. They also have a better prognosis compared to IDH-wildtype glioblastomas, with mean survival rates being about twice as long as IDH-wildtype glioblastomas [3]. Overall, high-grade gliomas (grade III-IV IDH-mutant astrocytomas/grade IV IDH wildtype glioblastomas) are very malignant and rapidly progressive, generally presenting with headaches, seizures, and focal neurological deficits (e.g. motor weakness, memory loss, and personality changes). Gliomatosis is an uncommon and extensively infiltrating type of glioma, which by definition, involves at least three contiguous brain lobes [4]. Occasionally, high-grade gliomas have the ability to infiltrate subarachnoid spaces, causing leptomeningeal gliomatosis and producing symptoms of back pain, myelopathy, radiculopathy, and cauda equina [5]. In very rare instances, high-grade intracranial gliomas have been seen infiltrating the spinal cord, resulting in spinal gliomatosis. Manifestations of spinal gliomatosis are non-specific - symptoms of spinal cord metastasis (weakness, sensory changes) [6] and infection (fever, seizures) [7,8], are all possible presentations. In the present case, the radiographic progression of a patient with IDH-mutant intracranial astrocytoma who subsequently developed severe spinal gliomatosis will be reviewed.

## Case presentation

**Figure 1:**
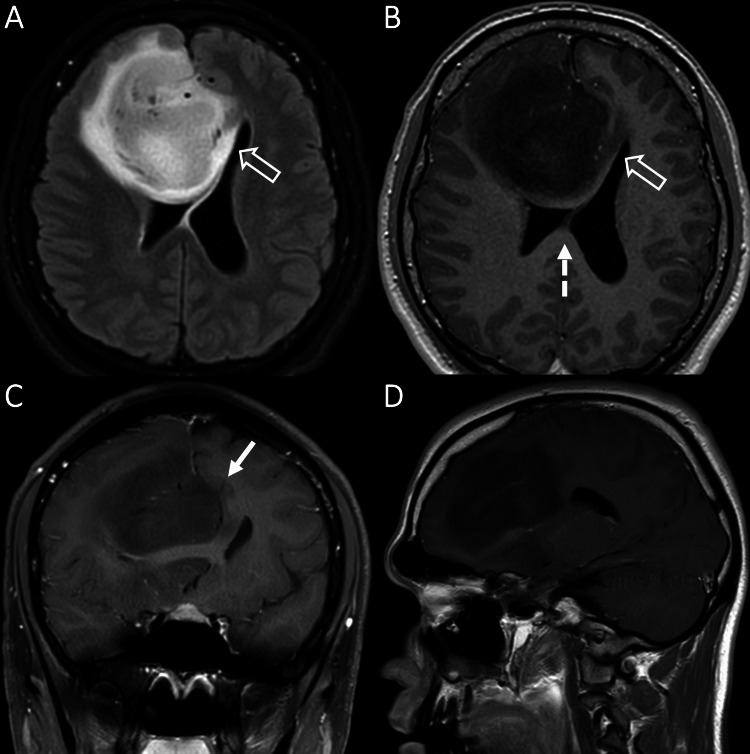
The patient’s baseline MRI showing a large right frontal oligodendroglioma, WHO grade III A, an axial MRI image showing a 6.2 cm solid, FLAIR, hyperintense mass centered at the right frontal pole. T1 post-contrast MRI images in B-D (B axial, C coronal, D sagittal) demonstrate T1 hypointensity with no significant enhancement. The mass causes subfalcine herniation (solid arrow in C) and there is an extension of white matter signal abnormality across the corpus callosum, across the midline to the left (open arrows in A, B). FLAIR: fluid-attenuated inversion recovery

An otherwise healthy, 18-year-old male presented to the clinic with a six-month history of absence seizure episodes and a witnessed grand mal seizure. Magnetic resonance imaging (MRI) at that time showed a large, 6.2 cm, heterogeneous, non-enhancing mass centered within the right anterior frontal lobe crossing midline to the left via the corpus callosum, with the anterior cerebral artery (ACA) running through the mass, as seen in Figure 1.

He was referred to the neurosurgery service. The patient underwent his first bi-frontal craniotomy under general anesthesia three days later with near-total resection of the tumor. Subsequent pathology results were significant for grade III oligodendrioma (alpha-thalassemia/mental retardation, X-linked (ATRX) mutant, IDH mutant, 1p/19q co-deletion, MIB1 index 4.1%). Postoperatively, he was referred to oncology, however, chemoradiation was declined in hopes of managing the disease using naturopathic methods.

**Figure 2:**
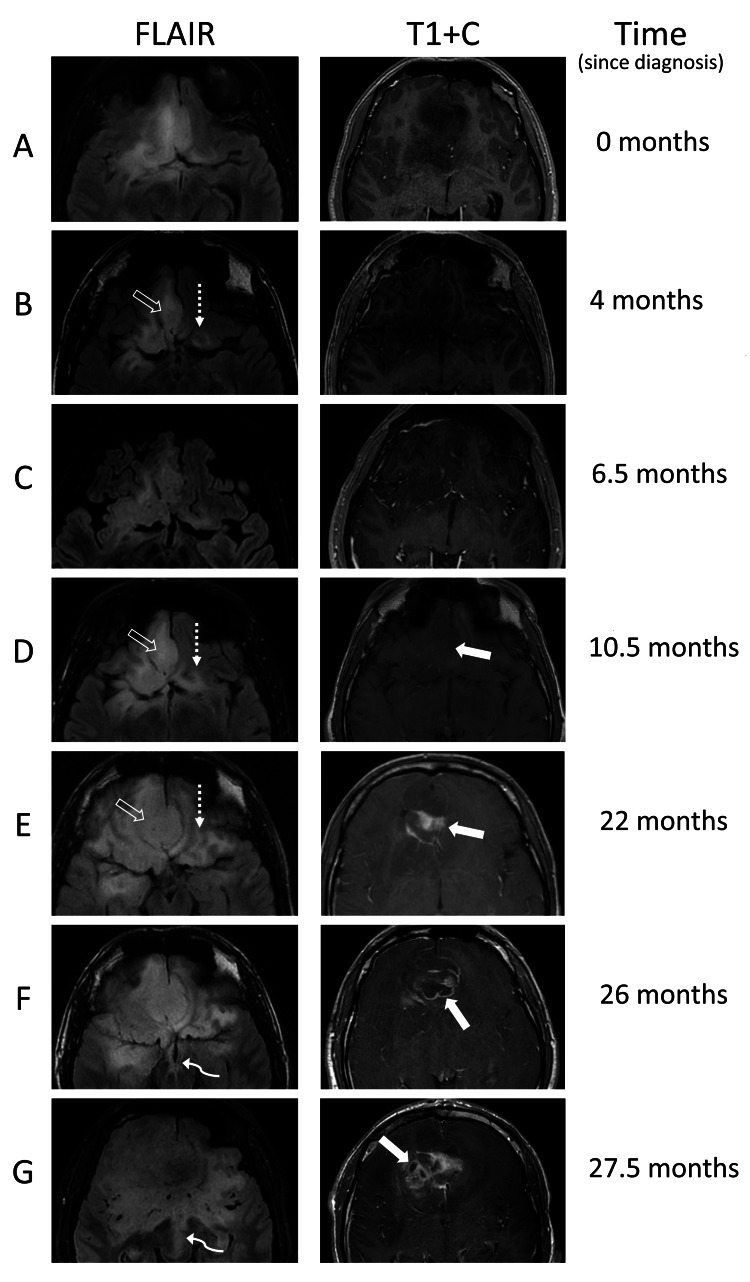
Axial MRI images showing subtle progression of gliomatosis throughout the brain over time A is the patient’s preoperative baseline scan demonstrating a nonenhancing, infiltrating glioma at the inferior aspect of the dominant frontal mass, into the bilateral inferior frontal lobes, right medial temporal lobe, and hypothalamus. B and C demonstrate no evidence of significant change in the immediate 2.5-month follow-up exam, however, disease progression can be better seen over longer periods of time, such as open arrows in B, D, and E, showing increased fullness and mass effect in the right gyrus rectus. Dashed arrows in B, D, and E show the same phenomenon of progressive gliomatosis in the left orbitofrontal gyri. Curved arrows in F and G show the progression of gliomatosis into the midbrain. Solid arrows in D and E demonstrate interval enhancement of the tumor, indicating progression into a higher-grade tumor, with the eventual development of necrotic areas as evidenced by the solid arrows in F and G. FLAIR: fluid-attenuated inversion recovery

Over the next two years, the patient continued to decline chemoradiation therapy but continued to receive serial follow-up MRIs. Serial MRI studies demonstrate continuous enlargement of residual tumor, progression of gliomatosis into the bilateral frontotemporal lobes, deep gray structures, and periventricular white matter, as well as enlarging enhancing regions of disease with necrosis, seen in Figure 2.

Clinical symptoms worsened with headaches and seizures, and 26 months after the first craniotomy, the patient underwent his second bi-frontal craniotomy for additional tumor resection. Subsequent pathology revealed a grade IV IDH1 mutant-glioblastoma, grade IV (post 2021 WHO classification now classified as an IDH1 mutant-astrocytoma, grade IV), with an MIB1 index of 30%. Postoperatively, the patient agreed to radiation therapy (60Gy/30Fx to the right frontal boost) and temozolomide therapy for eight weeks.

**Figure 3:**
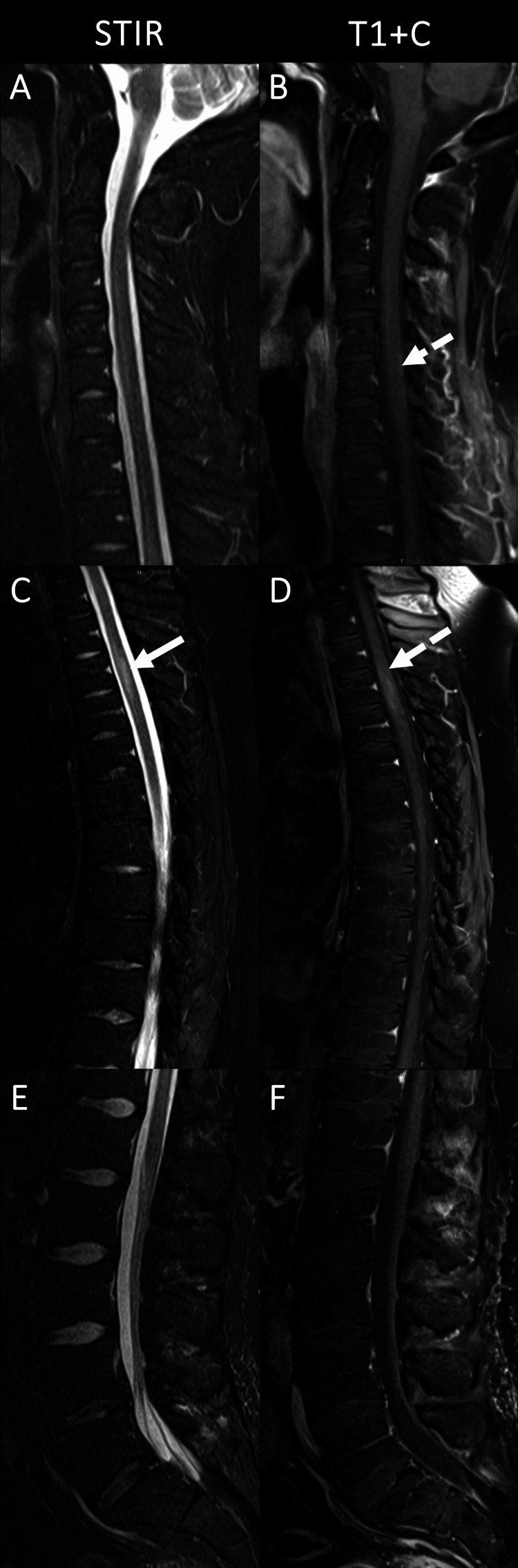
Sagittal MRI images of the spine taken 34 months after diagnosis, obtained for new bilateral lower extremity weakness. The solid arrow in C shows STIR hyperintensity within the left hemicord at T3-4 over a 2.2cm craniocaudal course. The dashed arrows in B and D show subtle enhancement within the cervical and thoracic spinal cord. Cauda equina nerve roots remain unremarkable.

Thirty-four months after the initial diagnosis, the patient developed dizziness and hydrocephalus-induced ataxia, requiring ventriculoperitoneal (VP) shunt placement. He also experienced new bilateral lower extremity weakness (strength 4/5 in bilateral hip flexors, knee extension/flexion, and plantar/dorsiflexion) and worsening constipation. MRI of the spine demonstrated a 2 cm expansile, T2, hyperintense lesion in the upper thoracic spine concerning for the development of spinal gliomatosis, as seen in Figure 3.

Palliative spinal radiation therapy in five fractions was pursued in an effort to relieve symptoms. A percutaneous endoscopic gastrostomy (PEG) tube was placed to maintain adequate nutrition.

**Figure 4:**
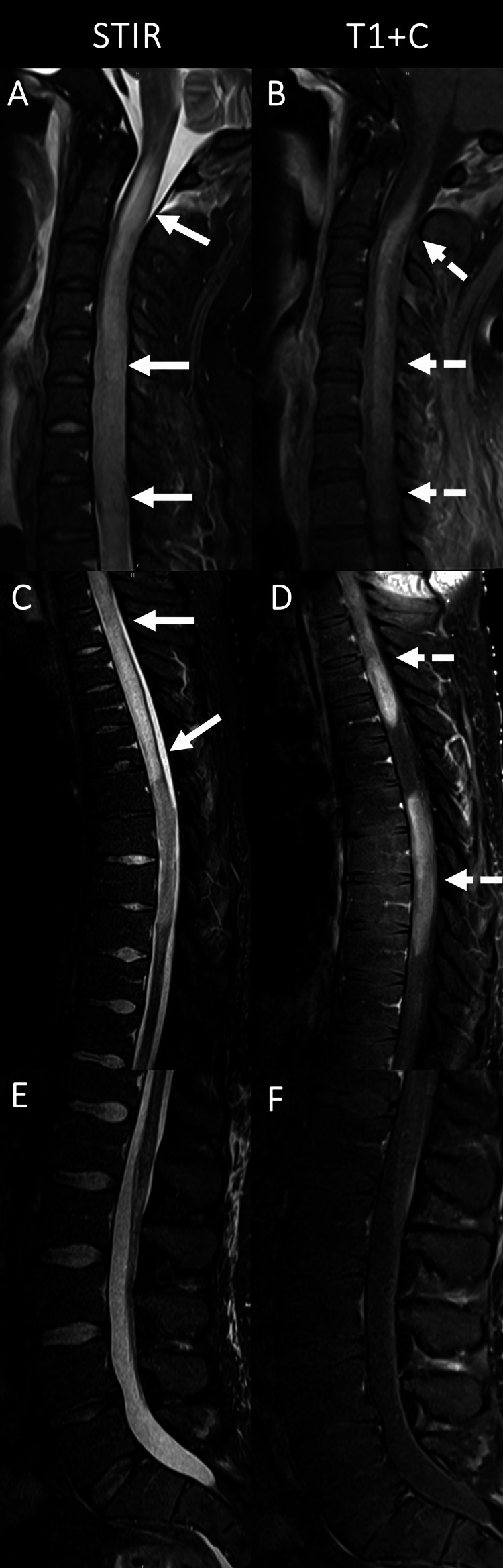
Sagittal MRI images of the spine obtained 38 months after diagnosis, four months after the MRI in Figure 3, showing marked progression of gliomatosis throughout the spinal cord Solid arrows in A and C show diffusely infiltrating expansile STIR hyperintense spinal cord lesions spanning the C1-T10 levels. B demonstrates ill-defined diffuse enhancement throughout the cervical cord (dashed arrows) while D demonstrates several more focal regions of the long segment and masslike enhancement within the thoracic cord (dashed arrows). The lumbar spine remains unremarkable. STIR: short tau inversion recovery (STIR)

Thirty-eight months after the initial diagnosis, the patient presented to the emergency department for shortness of breath after vomiting. He subsequently developed acute hypoxic respiratory failure requiring rapid sequence intubation to maintain oxygenation. His condition was further complicated by aspiration pneumonia secondary to Klebsiella pneumoniae and small bowel obstruction from stool impaction. On repeat brain and spinal MRI, there was evidence of marked disease progression with diffusely infiltrating expansile T2 short tau inversion recovery (STIR) hyperintense severe gliomatosis infiltrating the spinal cord from C1-T10, as shown in Figure 4.

Ultimately, 39 months after the initial diagnosis of the intracranial tumor and four months after the onset of spinal cord symptoms with spinal gliomatosis on imaging, the patient passed away due to septic shock.

## Discussion

This case demonstrates the natural progression of a high-grade diffuse glioma. Although the patient underwent surgical resection soon after diagnosis, chemoradiation was declined. Serial MRI demonstrated slow and continuous progression of gliomatosis, which is the expected outcome of an unchecked infiltrating glioma but is rarely documented so thoroughly and sequentially as in this case.

This case also illustrates the relevance of recent 2021 changes in the WHO classification scheme [2], which emphasizes that gliomas with IDH mutations are less aggressive despite the presence of aggressive histologic findings. Our patient’s grade III oligodendroglioma eventually transformed into a grade IV tumor, seen by the enhancement at 22 months on MRI, and pathologically defined after the second craniotomy as glioblastoma under the old classification scheme. However, the patient’s relatively long overall survival of 38 months is inconsistent with the poor survival expected of glioblastoma and more consistent with a grade IV astrocytoma, confirming the utility of the changes to the WHO classification scheme. Of note, prognostic factors such as tumor location, age of diagnosis, and histologic and molecular genetic factors affect survival as well. Walid found that age less than 40 as with our patient and combination therapy of surgery and chemotherapy/radiation, which our patient declined, prolonged survival, while an elevated MIB1 index as with our patient results in lower survival time [9].

The WHO classification system emphasizes other glioma molecular markers besides IDH status. Our patient’s initial molecular pathology was positive for ATRX mutant and tumor protein 53 (TP53) mutations. ATRX mutations are associated with IDH and TP53 mutations in astrocytomas found in the young adult group, especially for tumors found in the frontal cortex, all concordant with our patients’ course. ATRX mutations are more unstable, genetically resulting in an impaired DNA damage response; this is a good prognostic indicator for patients, as it makes the tumor more susceptible to chemotherapy, improving survival [10].

Our patient’s progression of gliomatosis into the spinal cord is a rarely seen finding. Given the relatively new WHO classification scheme differentiating grade IV glioblastoma and astrocytoma based on IDH mutation status, it is difficult to resolve the rate of spinal metastases between the two conditions. It is possible that differences in survival between these two entities may be a factor in how often spinal metastases are seen. Birbilis et al. previously described spinal metastases in 23 patients with primary intracranial glioblastoma multiforme over a 27-year period (1980-2007) [11]. Overall, spinal metastasis carried an extremely poor prognosis with a 100% case-fatality rate. Additionally, the progression from glioblastoma diagnosis to spinal metastasis was 14 months on average, and from spine metastasis to death was two to three months on average. Interestingly, Mariniello et al. found that the spread of primary intracranial tumors through cerebrospinal fluid (CSF) occurs in less than 0.01% of all primary CNS tumors, causing new-onset spinal symptoms [6]. Our patient had neurogenic bowel and lower extremity weakness. Upon comparison to patients from Biribillis’ study, lower extremity weakness appears to be a common symptom [11]. However, only one patient of the 23 had bowel dysfunction. In addition, as with our patient, almost every patient in that study had multiple levels of spinal involvement and received palliative radiation therapy. Some patients also received decompressive laminectomies. Our patient was not a candidate for laminectomy, as he was deemed too critically ill to undergo surgery.

Despite its rarity, it is imperative to consider spinal gliomatosis in a patient with a primary intracranial tumor with new-onset spinal symptoms (e.g. pain, radiculopathy, urinary/bowel incontinence, weakness, etc.) and rule out CSF-mediated drop metastasis into the spinal cord. While this condition has only been reported in 0.01% of primary intracranial tumors [6], an absence of routine screening guidelines may lead to a missed diagnosis in some cases, as the Response Assessment in Neuro-Oncology (RANO) Working Group does not address screening the spine [12]. This speaks to the limitations of this study, as the prevalence of spinal gliomatosis might be falsely low due to the lack of spinal screening.

## Conclusions

The natural progression of diffuse glioma is gliomatosis throughout the brain. Albeit rare, when a patient with a known primary intracranial tumor begins to develop spinal symptoms, such as pain, motor, or sensory changes, spinal gliomatosis should be an important complication to consider and investigation with spinal MRI is crucial. MRI imaging of the brain may be relatively stable while gliomatosis in the spine progresses unchecked if not seen. Unfortunately, the prognosis of spinal gliomatosis appears dismal.
